# Genistein decreases the breast cancer stem-like cell population through Hedgehog pathway

**DOI:** 10.1186/scrt357

**Published:** 2013-12-11

**Authors:** Panhong Fan, Shujun Fan, Huan Wang, Jun Mao, Yu Shi, Mohammed M Ibrahim, Wei Ma, Xiaotang Yu, Zhenhuan Hou, Bo Wang, Lianhong Li

**Affiliations:** 1 Department of Pathology, Dalian Medical University, Dalian 116044, P.R. China; 2 The Key Laboratory of Tumor Stem Cell Research of Liaoning Province, Dalian Medical University, Dalian 116044, P.R. China; 3 Department of Pathology, Thomas Jefferson University Hospital, Philadelphia, PA 19107, USA

## Abstract

**Introduction:**

The existence of breast cancer stem-like cells (BCSCs) has profound implications for cancer prevention. Genistein, a predominant isoflavone found in soy products, has multiple robust anti-tumor effects in various cancers, especially in the breast and prostate cancer. In this study, we aimed to evaluate genistein inhibition of BCSCs and its potential mechanism by culturing MCF-7 breast cancer cells and implanting these cells into nude mice.

**Methods:**

Cell counting, colony formation and cell apoptosis analysis were used to evaluate the effect of genistein on breast cancer cells’ growth, proliferation and apoptosis. We then used mammosphere formation assay and CD44CD24 staining to evaluate the effect of genistein on BCSCs *in vitro*. A nude mice xenograft model was employed to determine whether genistein could target BCSCs *in vivo*, as assessed by real-time polymerase chain reaction (PCR) and immunohistochemical staining. The potential mechanism was investigated utilizing real-time PCR, western blotting analysis and immunohistochemical staining.

**Results:**

Genistein inhibited the MCF-7 breast cancer cells’ growth and proliferation and promoted apoptosis. Both *in vitro* and *in vivo* genistein decreased breast cancer stem cells, and inhibited breast cancer stem-like cells through down-regulation of the Hedgehog-Gli1 Signaling Pathway.

**Conclusions:**

We demonstrated for the first time that genistein inhibits BCSCs by down-regulating Hedgehog-Gli1 signaling pathway. These findings provide support and rationale for investigating the clinical application of genistein in treating breast cancer, and specifically by targeting breast cancer stem cells.

## Introduction

Breast cancer remains one of the most commonly diagnosed cancers in the world. In Asian countries, particularly China and Japan, presumably due to diet and other environmental factors, the incidence of breast cancer is significantly lower than that in the western countries. Recent epidemiological and experimental studies have provided convincing evidence that genistein in soy-rich food contribute to the lower breast cancer incidence [1-3]. Genistein (4,5,7-trihydroxyisoflavone), a natural isoflavone phytoestrogen found in soybeans, has multiple functions on antitumor effects [4]. Additionally, it has been suggested that genistein can overcome cancer drug resistance and inhibit cancer relapse/recurrence [4,5].

Many human tumors contain cancer stem cells, which possess the self-renewal capacity, chemoresistance and an enhanced tumorigenicity [6]. In fact, the concept that cancers arise from stem cells or germ cells was first proposed almost 150 years ago. Cancer stem cells are now thought to play key roles in cancer growth, metastasis, and relapse. There has been compelling evidence that many cancer types, including breast cancer, are initiated from and maintained by a small population of cancer stem cells [7,8]. Breast cancer stem-like cells (BCSCs) are a small population of mostly resting cells defined by their long life, high clonogenicity, self-replicating potential, plasticity, and drug resistance [9]. A novel breast cancer combined chemotherapy and radiotherapy strategy that targets BCSCs has received extensive attention.

Researchers have found that several dietary compounds are promising chemoprevention agents against cancer stem cells, such as sulforaphane and curcumin [10,11]. Montales and colleagues were the first to demonstrate that genistein inhibition of mammosphere formation was mimicked by the Akt inhibitor perifosine and was associated with enhanced tumor suppressor phosphatase and tensin homolog deleted on chromosome 10 (PTEN) expression [12]. Diets rich in fruits and vegetables are implicated in breast cancer risk reduction, yet underlying mechanisms are poorly understood. The deregulation of some pathways is well known to inhibit tumorigenesis involving cancer stem cell signaling pathways, such as Hedgehog, Notch, and Wnt/β-catenin in breast cancer [13]. The Hedgehog pathway regulates the embryonic development of both invertebrates and vertebrates, and contributes to the formation of different organs and tissues, including the neural tube. Three mammalian homologs of this gene were subsequently identified: Sonic hedgehog (Shh), Indian hedgehog and Desert hedgehog. Shh is the most widely studied and best characterized [14]. The receptor Patched (Ptch) is a 12-transmembrane protein that acts catalytically to inhibit the seven-transmembrane protein Smoothened (Smo), rendering the pathway inactive in the absence of Hedgehog ligand. Binding of Hedgehog ligand inactivates Ptch, derepressing Smo and resulting in positive Hedgehog pathway signaling. When Smo is inactive, a multiprotein complex constitutively processes the Gli proteins to short, transcriptionally repressive forms. The Gli family contains Gli1, Gli2, and Gli3, which share five highly conserved tandem C2H2 zinc finger domains and a histidine–cysteine linker sequence between the zinc fingers. In humans, Gli1 acts as an activator, Gli2 as an activator or as a repressor depending upon its catalytic, and Gli3 as a repressor [15-17].

In the present study, we evaluated the efficacy and mechanisms of genistein suppressing the population of BCSCs from MCF-7 human breast cancer cells by examining tumor growth *in vivo*, mammosphere formation *in vitro*, and Hedgehog pathway expression.

## Materials and methods

### Reagents

Genistein was obtained from Sigma-Aldrich (St Louis, MO, USA), and dissolved in dimethyl sulfoxide (DMSO) at different doses for the experiments. Equal treatment volumes of DMSO were used as a vehicle control. All other materials were of analytical grade and were obtained from commercial sources.

### Cell lines and cell proliferation assay

Human breast cancer cell line MCF-7 was purchased from the Cell Bank of Type Culture Collection of Chinese Academy of Sciences, Shanghai Institute of Cell Biology, Shanghai, China. The cells were respectively cultured in Dulbecco’s modified Eagle’s medium (HyClone, Logan, UT, USA) supplemented with 10% fetal bovine serum (HyClone). All cells were maintained at 37°C, in 5% carbon dioxide and 95% relative humidity.

For growth inhibitory studies, MCF-7 cells were seeded in 96-well plates at a density of 3 × 10^4^ cells/well. The cells were incubated with genistein at concentrations of 0, 2.5, 5, 10, 15, 20, 30, 50, and 70 μM for 48 hours. After adding the solution of the Cell Counting Assay Kit-8 (Sigma-Aldrich) to cells/well, the cells were incubated for another 2 hours. The absorbance was measured with a microplate reader at 450 nm. The amount of the formazan dye, generated by the activated dehydrogenases in cells, was directly proportional to the number of living cells. Addition of medium alone was used as the blank control group. To estimate the inhibitory rate of cell growth, the concentration that inhibits 50% of the growth of control cells was calculated. All experiments were performed three times independently.

### Colony formation assay

MCF-7 cells were treated with genistein at concentrations from 0 to 15 μM for 48 hours. The viable cells were counted and seeded for colony formation assay in six-well plates at 300 cells/well. During colony growth, the culture medium was replaced every 3 days. Colonies with over 50 cells were counted under an inverted microscope (Olympus, Japan) on day 7 after seeding, to calculate the formation rate:

$$\begin{array}{l} \mathrm{Colony}\;\mathrm{formation}\;\mathrm{rate}=\mathrm{number}\;\mathrm{of}\;\mathrm{colonies}/\mathrm{number}\;\\ \mathrm{of}\;\mathrm{seeded}\;\mathrm{cells}\;\times \;100\% \end{array}$$

Each experiment was carried out in triplicate.

### Cell apoptosis analysis

Cell apoptosis was analyzed by flow cytometry. Briefly, 1 × 10^6^ cells were collected and washed in phosphate-buffered saline after treatment with different concentration of genistein for 48 hours. After washing with phosphate-buffered saline, the cells were resuspended in 500 μl binding buffer and incubated with 5 μl fluorescein isothiocyanate (FITC)–Annexin-V (BD Biosciences, San Jose, CA, USA) and 10 μl propidium iodide (BD Biosciences) for 15 minutes at 4°C in the dark. Apoptosis was measured using flow cytometry to quantify the levels of phosphatidylserine on the outer membrane of apoptotic cells. The results were analyzed by flow cytometry using the BD FACS Aria cell sorter (BD, Franklin Lakes, NJ, USA). This experiment was repeated three times.

### Mammosphere formation assay

The mammosphere forming assay was performed as described previously with slight modification [18]. Briefly, the cells were plated in ultra-low-attachment six-well plates (Corning, Acton, MA, USA) at a density of 20,000 cells/ml in primary culture and 1,000 cells/ml in passages, which were supplemented with 2 mmol/l l-glutamine (ATCC, Manassas, VA), 2% B27 supplement (Invitrogen, Carlsbad, CA, USA), 20 ng/ml human recombinant epidermal growth factor (Sigma-Aldrich) and 20 ng/ml basic fibroblast growth factor (Sigma-Aldrich), 4 μg/ml heparin and 5 μg/ml insulin (Sigma-Aldrich). Mammospheres were counted after culture for 7 days under a Nikon Eclipse TE2000-S microscope (Japan) and photographs were acquired with Meta Morph.

### CD44 and CD24 staining

The CD44^+^ and CD24^–^ breast cancer cell population was reported previously to include BCSCs [19]. After treatment of genistein for 48 hours, the MCF-7 cells were stained with phycoerythrin-conjugated anti-human CD24 antibody (Invitrogen) and FITC-conjugated anti-human CD44 antibody (Invitrogen) according to the manufacturer’s instructions. Samples were analyzed using a FACS Calibur flow cytometer and Cell Quest software (BD).

### Tumor growth and morphologic analysis *in vivo*

All studies involving mice were approved by the Animal Care and Use Committee of Dalian Medical University (Dalian, China). Fifteen 6-week-old to 8-week-old female nude mice were purchased from the Experimental Animal Center of Dalian Medical University. Then 1 × 10^6^ MCF-7 cells were suspended in 100 μl phosphate-buffered saline mixed with matrigel (1:1) and injected into the mouse mammary fat pad. Two weeks after cell injection, the mice were randomly separated into three groups, which were intraperitoneally injected with control (0.1% DMSO solution) or with 20 and 50 mg/kg genistein respectively (dissolved in 0.1% DMSO solution) daily for 2 weeks. Tumors were measured with a caliper, and the volume was calculated:

Volume = 1/2 (width^2^ × length)

The tumors were excised, weighed, and frozen at −80°C until processing for RNA and protein isolation. For histological study, portions of tumors were fixed in 10% neutral-buffered formalin, were paraffin embedded, and then 4 μm sections were stained for immunohistologic assay.

### Immunohistochemical staining

The tumor sections were deparaffinized in xylene and rehydrated with graded ethanol followed by microwave heating for 30 minutes in 10 mM sodium citrate buffer (pH 7.2); 0.3% hydrogen peroxide solution was used for the blocking of endogenous peroxide activity. The primary monoclonal antibodies against aldehyde dehydrogenase isoform 1 (ALDH1; (Santa Cruz Biotechnology, Santa Cruz, CA, USA), human SMO (Santa Cruz Biotechnology), and Gli1 (Santa Cruz Biotechnology) were applied at 4°C overnight. The sections were incubated with horseradish peroxidase-labeled goat anti-mouse/rabbit antibody (Maixin Bio) for 30 minutes at room temperature. 3,3′-Diaminobenzidine was used as the chromogen and hematoxylin as the nuclear counterstain. The sections were dehydrated, cleared, and mounted.

### Western blotting analysis

For the western blot analysis, 1 × 10^6^ cells incubated with different concentrations of genistein for 48 hours were harvested and lysed. The protein concentration was determined by the Bradford method with bovine serum albumin (Sigma-Aldrich). Each sample was treated with anti-Smo (Sigma-Aldrich) or anti-Gil1 (Santa Cruz, Inc.) primary antibodies. Primary anti-bodies were detected by horseradish peroxidase-conjugated antibody (1:2,000; GE Healthcare). Signals were detected by the enhanced chemiluminescence detection system (Amersham Pharmacia Biotech).

### Real-time polymerase chain reaction

Total RNA was extracted from cell pellets using the Quick Prep total-RNA Kit (Amersham Biosciences, UK), according to the manufacturer’s instructions. Each sample was incubated for 48 hours with different concentrations of genistein. Reverse transcription was performed using a Taq Man Reverse Transcription Kit (Applied Biosystems). For quantitative real-time reverse transcription polymerase chain reaction, 1 ml gene primers with the SYBR Green RT-PCR Kit (QIAGEN, Valencia, CA, USA) in 20 ml reaction volume was applied. The relative changes in the amount of transcripts in each sample were determined by normalizing with the glyceraldehyde 3-phosphate dehydrogenase mRNA levels. Primers were designed as: ALDH1 (forward, 5′-AATGGCATGATTCAGTGAGTGGC-3′; reverse, 5′-GAGGAGTTTGCTCTGCTGGTTTG-3′), SMO (forward, 5′-CTTTGTCATCGTGTACTACGCC-3′; reverse, 5′-CGAGAGAGGCTGGTAGGTG-3′), Gli1 (forward, 5′-GAACCCTTGGAAGGTGATATGTC-3′; reverse, 5′-GGCAGTCAGTTTCATACACAGAT-3′), and glyceraldehyde 3-phosphate dehydrogenase (forward, 5′-CATGAGAAGTATGACAACAGCCT-3′; reverse, 5′-AGTCCTTCCACGATACCAAAGT-3′).

### Statistical analysis

Statistical analysis was done using SPSS software version 11.0. Statistical differences were determined using a two-tailed Student *t* test. The results were expressed as the mean ± standard deviation. *P*<0.05 was considered statistically significant.

## Results

### Genistein inhibits breast cancer cells

**Figure 1:**
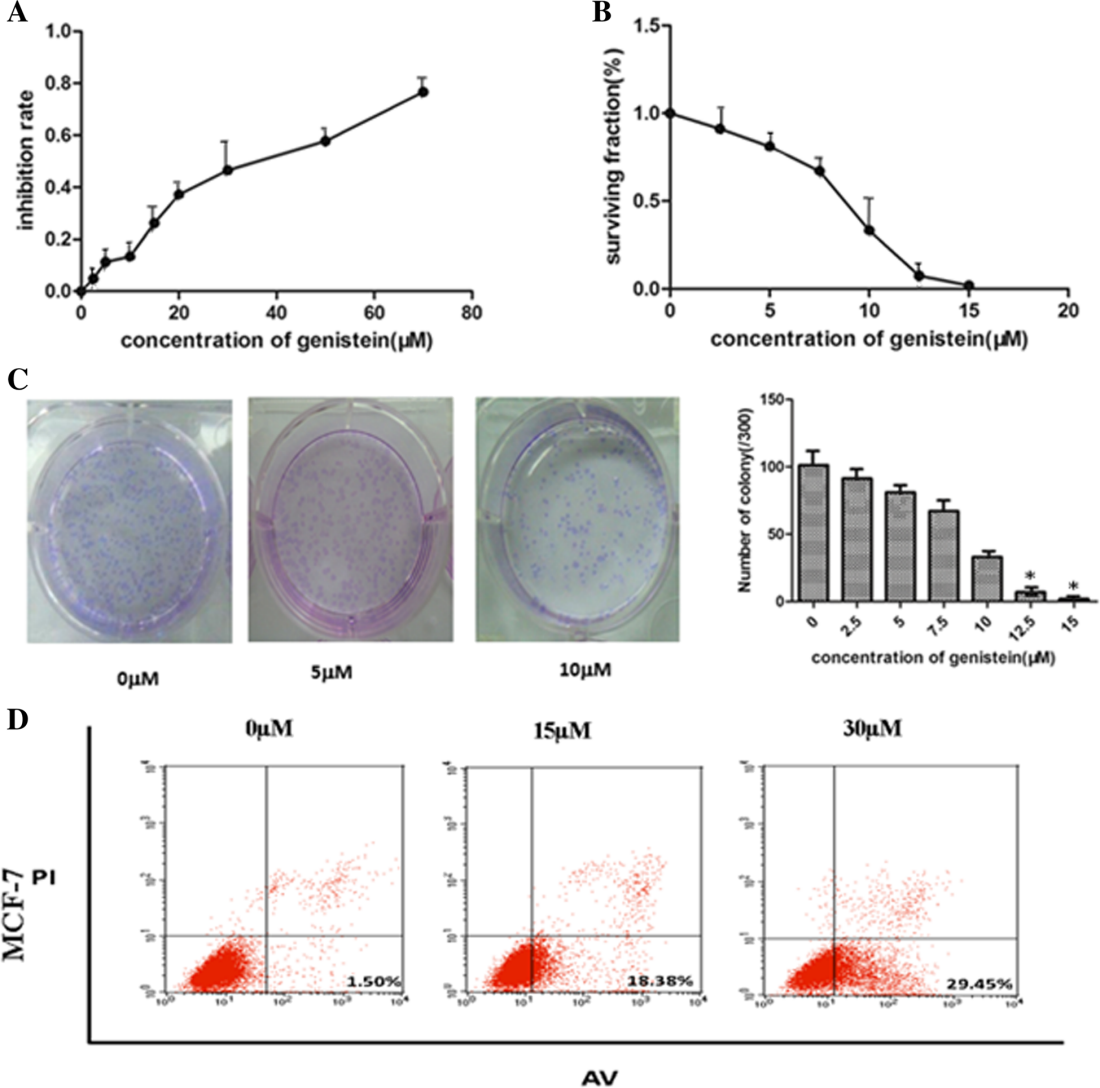
**Genistein inhibits breast cancer cells. (A)** MCF-7 cells growing in the log phase were treated with increasing concentrations of genistein for 48 hours. The anti-proliferation effect of genistein was measured by Cell Counting Kit-8 (CCK-8;) assay. **(B)**, **(C)** Genistein reduced the number of colonies in MCF-7 cells. Genistein’s effect on the surviving fraction on MCF-7 cells was detected by colony formation assay. The number of colonies was counted after 7 days. All data presented as mean ± standard deviation (*n* ≥3). **(D)** Genistein increased the percentage of late period apoptotic cells labeled with Annexin V– fluorescein isothiocyanate (FITC)/propidium iodide (PI) in MCF-7 cells. Experiments were repeated three times, and similar results were obtained. Representative scatter grams from flow cytometry profile represent Annexin V–FITC (AV) staining on the *x* axis and PI on the *y* axis. *P <0.05, Student’s *t* test. Each condition was repeated three times and error bars represent standard deviations.

We demonstrated the anti-proliferative effects of genistein in the human MCF-7 breast cancer cell line with the Cell Counting Kit-8 assay. The cells were treated with increasing concentrations of genistein for 48 hours, and the inhibitory effect was measured by the ratio of inactive cells to the control (Figure 1A). Inactive cells increased with elevated genistein concentration. The concentration that inhibits 50% of the growth of control cells at 48 hours post treatment was 32.5 μM. The genistein concentrations equivalent to the concentration that inhibits 50% of the growth of control cells were then used throughout the remainder of the study. Consistently the survival cells decreased as the genistein dosage increased (Figure 1B). The colony number was also reduced by treatment with elevated genistein concentration for 7 days compared with the control group (Figure 1C). Furthermore, exposure of cells to genistein for 48 hours resulted in an accumulation of apoptotic cells (Figure 1D). The induction of apoptosis was in a dose-dependent manner. Our results demonstrate that genistein had multiple effects on MCF-7 cell growth, proliferation, and apoptosis.

### Genistein suppresses breast cancer stem cells *in vitro*

**Figure 2:**
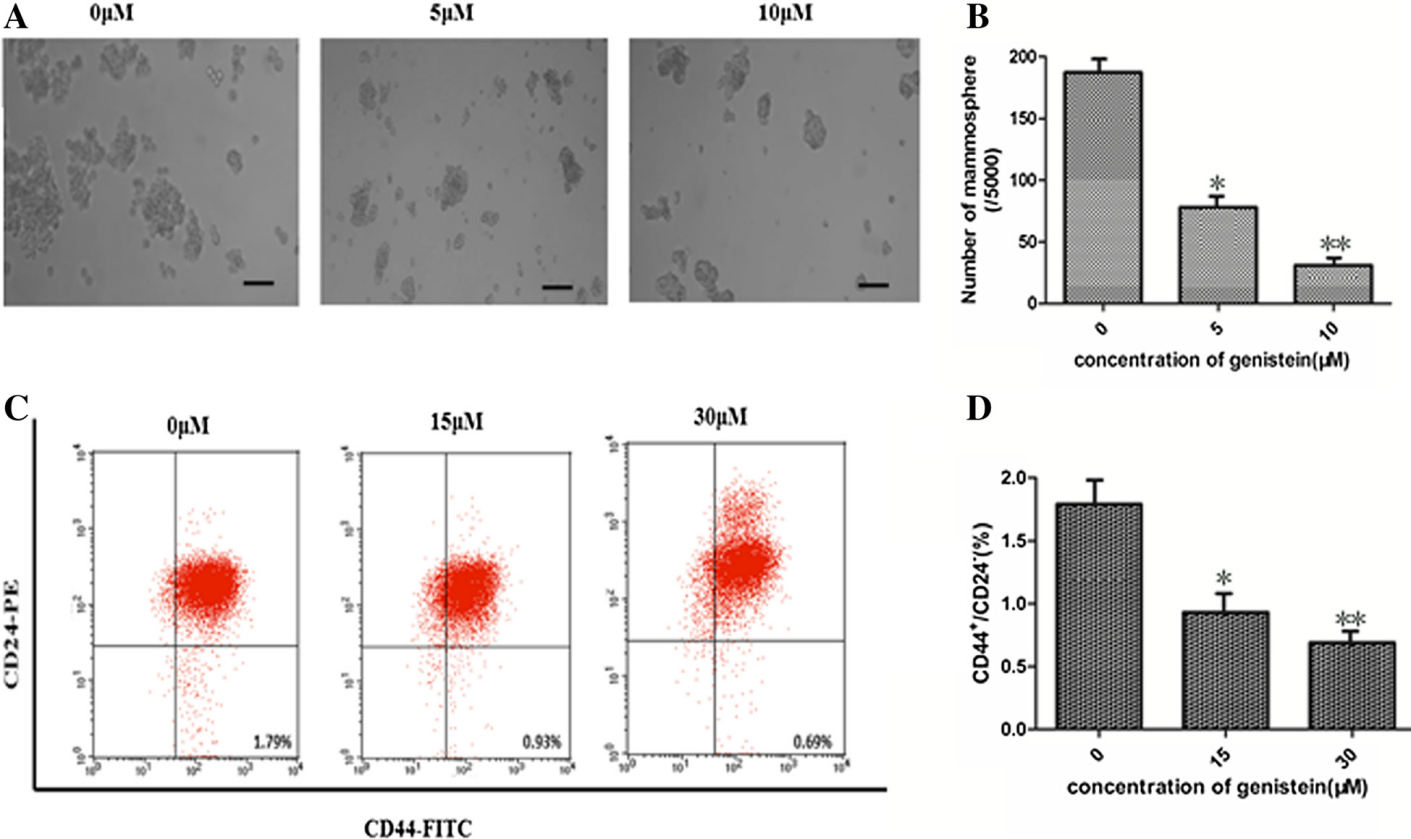
**Genistein decreases breast cancer stem cells *in vitro*. (A)**, **(B)** Genistein decreased the size **(A)** (×200, scale bar = 100 μm) and number **(B)** of mammospheres. MCF-7 cells were treated with different concentrations of genistein, and then were cultured in ultra-low-attachment plates with serum-free medium for 7 days. Mammospheres were collected and evaluated. **(C)**, **(D)** The percentage of CD44^+^/CD24^–^ breast cancer stem-like cells was reduced by genistein treatment. The phenotype of CD44^+^/CD24^–^ cells was measured by flow cytometry. Similar results were obtained in three independent experiments, and the representative microscopic picture and flow cytometry pattern are shown. *P <0.05, **P <0.01, Student’s *t* test. FITC, fluorescein isothiocyanate; PE, phycoerythrin.

To investigate effects of genistein on the size and number of the stem cell population, we performed the mammosphere formation assay in human MCF-7 breast cancer cells. BCSCs have been demonstrated to be enriched in nonadherent spherical clusters of cells, termed mammospheres [18], which in turn can give rise to the secondary spheres and differentiate into multiple lineages. After treatment with varying concentrations of genistein, the MCF-7 cells were harvested and subcultured for two passages in the absence of genistein. As shown in Figure 2A,B, genistein reduced both the number and size of mammospheres.

Both CD44^+^ and CD24^–^ have been used as specific markers to identify the BCSCs from human tumor tissues [19]. The CD44^+^CD24^–^ cell population is capable of self-renewal and generating tumors resembling breast cancer. However, there is no report of genistein effect on MCF-7 BCSCs. We evaluated the CD44^+^CD24^–^ cell population in MCF-7 cells with fluorescence-activated cell sorting after genistein treatment *in vitro*. As shown in Figure 2C,D, the CD44^+^CD24^–^ population in genistein-treated MCF-7 cells was significantly decreased by 62% (at 15 μM genistein) and 87% (at 30 μM genistein) respectively, compared with the control (*P* <0.05). These findings therefore demonstrate that genistein can suppress the BCSC population *in vitro*.

### Genistein reduces breast cancer stem cells *in vivo*

**Figure 3:**
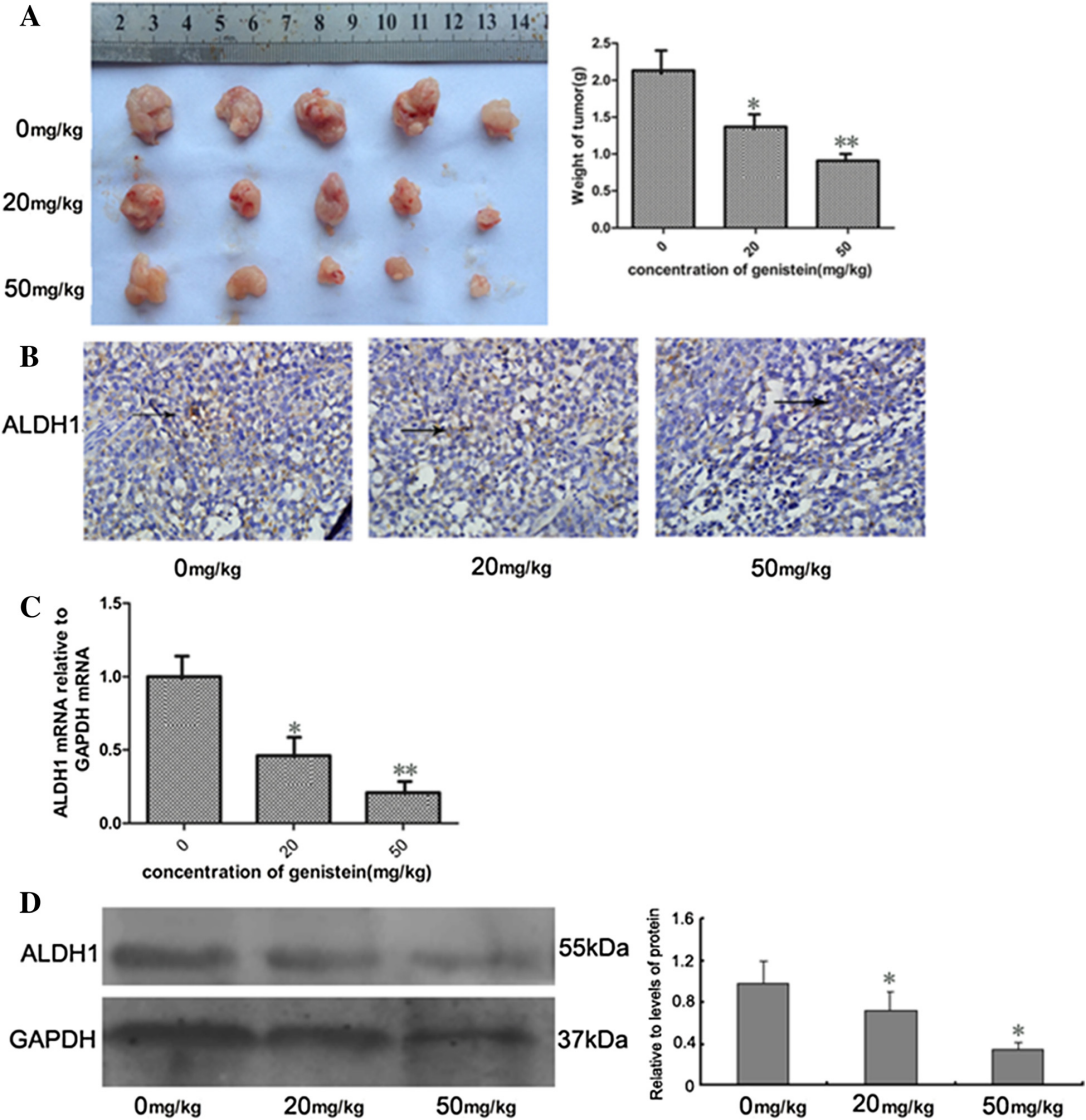
**Genistein decreased breast cancer stem cells *in vivo*. (A)** Genistein remarkably reduced the tumor volume and weight: 1 × 10^6^ MCF-7 cells were inoculated into the mouse mammary fat pad for 2 weeks, and then were treated daily with 20 and 50 mg/kg genistein. After 2 weeks of treatment, mice were sacrificed. Error bars represent standard deviations, *n* = 5. **(B)** Immunohistochemistry staining with aldehyde dehydrogenase isoform 1 (ALDH1) antibodies (brown in cytoplasm), ×200. **(C)** Real-time polymerase chain reaction analyses of ALDH1 in the mouse tumor tissues. **(D)** ALDH1 protein level before and after treatment with genistein. * *P* <0.05, **P <0.01, Student’s *t* test. GAPDH, glyceraldehyde 3-phosphate dehydrogenase.

Many studies have suggested that cancer stem cells may contribute to the development of chemoresistance. To determine whether genistein could have an effect on BCSCs *in vivo*, we utilized a xenograft model of MCF-7 cells in nude mice. Two weeks after cell inoculation, animals were randomly divided into three groups to receive daily intraperitoneal injection of 0.1% DMSO solution only (control group) or 20 and 50 mg/kg genistein (dissolved in 0.1% DMSO solution). After 2 weeks of treatment, the grafted tumors were dissected and weighed. In comparison, the average tumor weights in genistein-treated mice were 46% and 68% of that in control animals (*P* <0.05) (Figure 3A).

Since studies have shown that breast cancer cells with high aldehyde dehydrogenase (ALDH) activity have enriched tumorigenic stem cells [20], we examined the ALDH levels in the tumors isolated from the three groups by immunohistochemical staining and real-time polymerase chain reaction. Genistein significantly reduced ALDH staining (Figure 3B), mRNA expression (Figure 3C), and protein level (Figure 3D) by more than 50% compared with that from control mice (*P*<0.05). These results suggest that genistein was able to target BCSCs to reduce the xenograft tumors.

### Genistein inhibits breast cancer stem cells through downregulation of the Hedgehog–Gli1 signaling pathway

**Figure 4:**
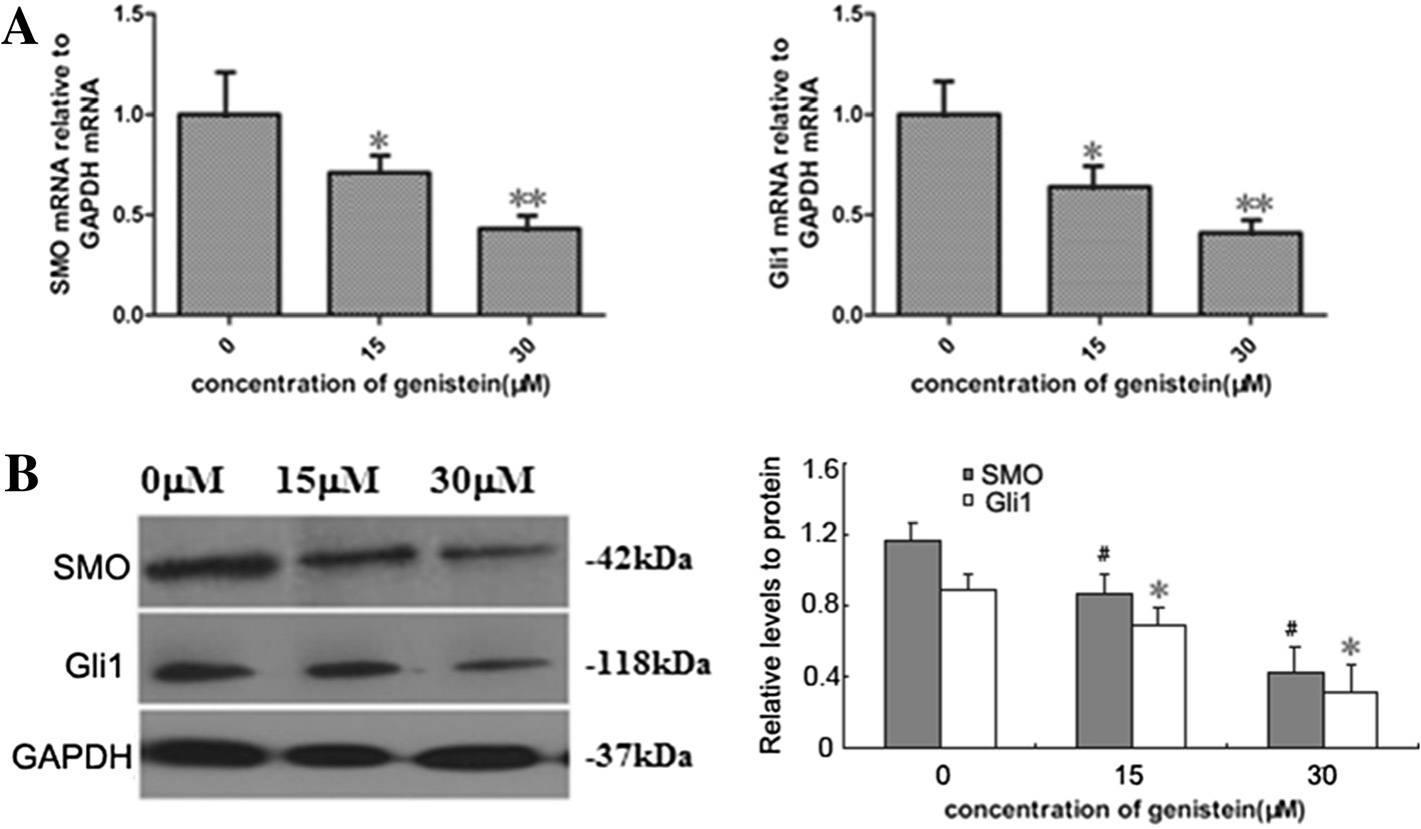
**Hedgehog–Gli1 pathway inhibition by genistein *in vitro*. (A)** Real-time polymerase chain reaction analysis of human Smoothened (SMO) and Gli1 in MCF-7 cells. The cells were incubated with different concentrations of genistein for 48 hours. **(B)** Protein levels of SMO and Gli1 were measured by western blot analysis in MCF-7 cells and accompanied by a quantitative bar chart. As an internal control, glyceraldehyde 3-phosphate dehydrogenase (GAPDH) was used for normalization. *P <0.05, ^#^*P* <0.05, **P <0.01, Student’s *t* test.

We next investigated the mechanisms underlying the inhibitory effects of genistein on BCSCs. The Hedgehog pathway is known to be an important regulator of stem cell self-renewal. Emerging data from many human tumors have suggested that Hedgehog–Gli1 signaling regulates cancer stem cells [21]. Aberrant activation of SMO and Gli1 are known as the key process of the Hedgehog–Gli1 signaling pathway. As shown in Figure 4A, 30 μM genistein significantly decreased the mRNA level of Smo by 57% and of Gli1 by 59% in MCF-7 cells compared with control. This was further confirmed with western blot analysis (Figure 4B).

**Figure 5:**
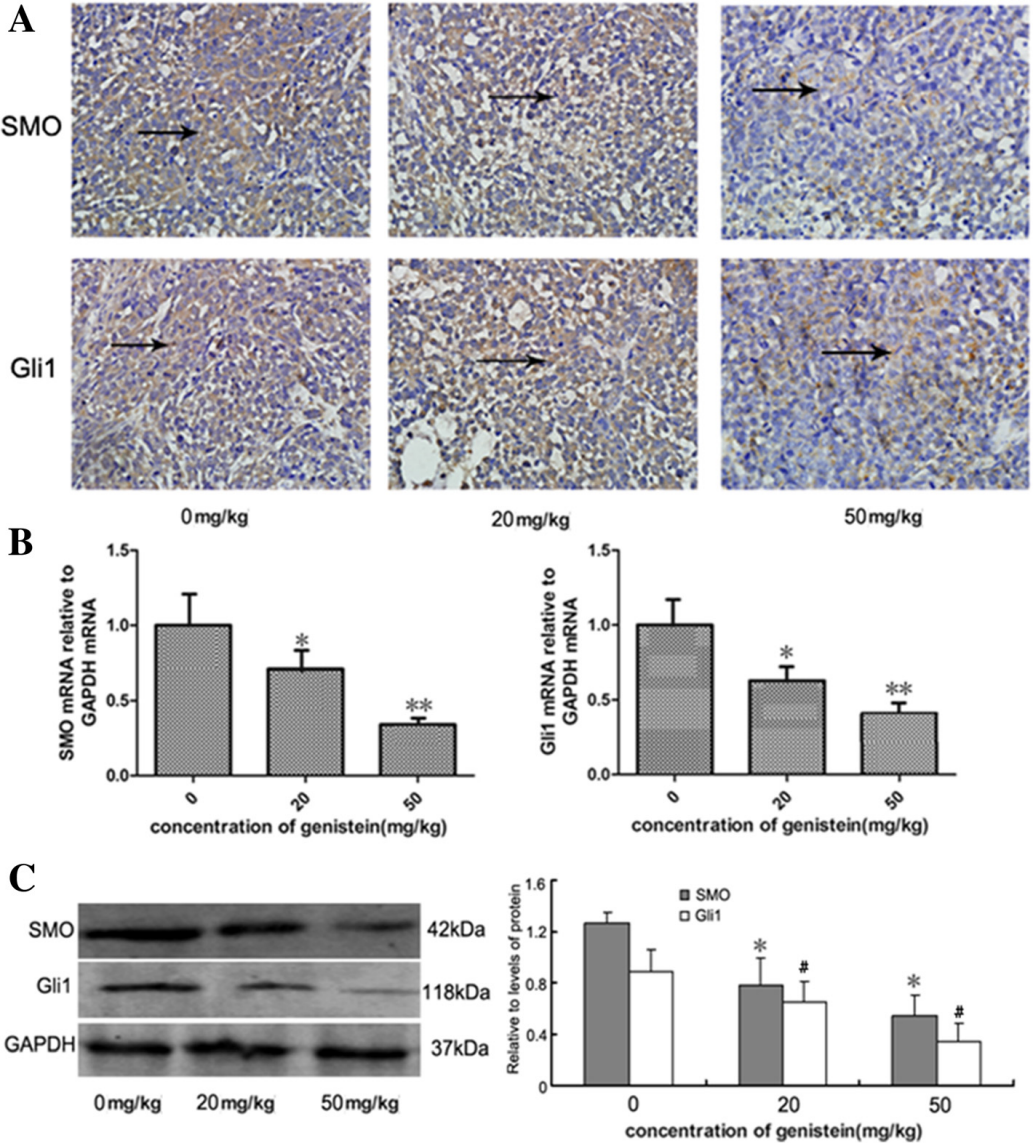
**Hedgehog–Gli1 pathway inhibition by genistein *in vivo*. (A)** Immunohistochemistry staining with human Smoothened (SMO) and Gli1 antibodies (brown in cytoplasm), ×200. **(B)** Real-time polymerase chain reaction analysis of SMO and Gli1 in mice tumors. **(C)** Smo and Gli1 protein level in tumor tissue and accompanied by a quantitative bar chart. Data presented as mean ± standard deviation, *n* = 5. *P <0.05, **P <0.01, Student’s *t* test. Each condition was repeated three times and error bars represent standard deviations. GAPDH, glyceraldehyde 3-phosphate dehydrogenase.

To further investigate whether the Hedgehog–Gli1 pathway was inhibited by genistein *in vivo*, we examined mRNA levels and protein expression of Smo and Gli1 from the tumors yielded from control and genistein-treated mice. As shown in Figure 5A, the genistein treatment significantly decreased the Smo-positive and Gli1-positive staining in breast tumor tissues, compared with the control group. Consistently, the mRNA level and protein level of Smo and Gli1 also significantly decreased after genistein treatment (Figure 5B,C). In summary, these results suggest that genistein suppressed BCSCs by downregulating the Hedgehog–Gli1 pathway.

## Discussion

In this study, we for the first time demonstrated that genistein has multiple anti-breast cancer cell effects, not only suppressing proliferation and inducing apoptosis of the cancer cells, but also specifically inhibiting the cancer stem cells by downregulating the Hedgehog–Gli1 pathway.

Cancer stem cells are currently believed to be the root cause of cancer, which contribute to resistance to therapy, tumor recurrence, and distant metastasis. However, lack of efficacy of current chemotherapies in advanced and metastatic disease requires novel approaches to specifically target the cancer stem cell population [22,23]. Therapies directed against both differentiated cancer cells and cancer stem cells may thus provide advantages to treat these diseases. The well-known curcumin and sulforaphane were shown to interfere with Wnt signaling pathway in cancer cells [10,11,17]. Some agents such as sulforaphane, which regulates the Wnt/β-catenin signaling pathway, have been shown to reduce the BCSC population [10]. For the past few years, studies have focused on efficacy of genistein, a predominant isoflavone found in soy products, in various cancers, especially in the breast and prostate cancer. Genistein is a well-characterized multifunctional soy isoflavone with biological activities including protein tyrosine kinase inhibition, estrogen receptor activation, and antioxidant activity [15,24]. We therefore focus on the relationship between genistein and BCSCs and the potential mechanism involved.

We first tested whether genistein has a killing effect on MCF-7 breast cancer cells. Not surprisingly, genistein effectively shows multiple anti-cancer effects on proliferation, activation, and apoptosis. To study the cancer stem cells, we decided to use mammosphere culture that was first used to isolate and expand mammary stem/progenitor cells by Dontu and colleagues [18], based on the ability of stem cells to grow in serum-free suspension, while differentiated cells fail to survive under the same condition [25]. Using this technique, we demonstrated that genistein (5 to 10 μM) significantly reduced both the size and number of mammospheres formed by MCF-7 cells. Another technique is to use cell markers to distinguish BCSCs from differentiated cancer cells. As few as 200 CD44^+^CD24^−/low^lin^−^ cells have been reported to be able to generate a breast tumor [19]. We thus utilized a CD44 and CD24 staining flow cytometry assay to evaluate the ability of genistein to target BCSCs. We demonstrated that genistein specifically suppressed the CD44^+^CD24^–^ cell population in MCF-7 cells. These findings support that genistein is effective in reducing BCSCs *in vitro*.

The injection of human breast cancer cells into the mammary fat pad of nude mice provides a reliable and sensitive *in vivo* system for studying human breast cancer. We therefore tested whether genistein was able to target BCSCs *in vivo* by using this xenograft model. Daily injection of genistein for 2 weeks successfully suppressed tumor growth in nude mice. We also examined ALDH1 in these animals treated with or without genistein. ALDH is another important marker for BCSCs. In a previous study, 50,000 ALDH-negative cells failed to form tumors, while <500 ALDH-positive cells were able to generate a breast tumor within 40 days [20]. We observed in genistein-treated tumor that the ALDH protein and mRNA levels were significantly lower than those in control group mice. These are consistent with the *in vitro* observation that genistein specifically targeted BCSCs. The capacity of genistein in killing BCSCs may be significant for chemoprevention.

The Hedgehog gene was first discovered by Nusslein Volhard and Wieschaus in *Drosophila melanogaster* larvae, and has been shown critical for the self-renewal of many cancer stem cells [26,27]. This signaling pathway can be a simple conclusion of the Hedgehog–Ptch1–Smo–Gli process. Gil1, which is independent of Smo activation, is an important regulator of the effect of the Hedgehog pathway on transcription. The proliferation of cancer stem cells could be inhibited by a blockade of the Hedgehog pathway due to the deletion of SMO or (and) Gli1. In this study, we observed the reduced expression of SMO and Gli1 after treatment with genistein both *in vitro* and *in vivo*. In the presence of genistein, downregulation of the Hedgehog pathway may contribute to the loss of stemness of BCSCs. This warrants further studies (such as rescue experiment by overexpression of SMO and Glil) to establish the conclusively causative role of this downregulation in the inhibition of BCSCs by genistein.

## Conclusion

We demonstrate for the first time that genistein specifically inhibits BCSCs, in association with downregulation of the Hedgehog–Gli1 self-renewal pathway. Genistein, a natural compound, has no reported toxic side effects, and may provide a new safe and effective therapeutic way for the treatment of breast cancer. This study thus provides a strong rationale for investigating the chemoprevention entity of genistein in clinical trials.

## Abbreviations

ALDH: aldehyde dehydrogenase; ALDH1: aldehyde dehydrogenase isoform 1; BCSC: breast cancer stem-like cell; DMSO: dimethyl sulfoxide; FITC: fluorescein isothiocyanate; Ptch: Patched; Shh: Sonic hedgehog; Smo: Smoothened.

## Competing interests

The authors declare that they have no competing interests.

## Authors’ contributions

PF conceived of and performed all the experiments, analyzed the data, and drafted the manuscript. SF conceived of and performed animal experiments, analyzed the data and revised the manuscript. HW was responsible for the molecular biology experiments. JM performed the cell experiments and analyzed the data. YS revised the manuscript. MMI analyzed the data. BW contributed essential reagents and tools. WM performed animal experiments. XY analyzed the data. ZH analyzed the data. LL conceived of the research, coordinated the study, interpreted the data, and revised the manuscript. All authors read and approved the final manuscript.
